# High-efficiency femtosecond laser fabrication of graphene-hybrid planar micro-supercapacitors with micro/nanostructured electrodes

**DOI:** 10.1038/s41377-025-02182-5

**Published:** 2026-01-21

**Authors:** Yuyuan Zhang, Tingting Zou, Haobo Jiang, Xiuyan Fu, Wei Xin, Yiyang Meng, Xilin Li, Jun-Ming Cao, Lin Yang, Yuanzheng Li, Weizhen Liu, Dongdong Han, Xing-Long Wu, Jianjun Yang, Haiyang Xu, Yichun Liu

**Affiliations:** 1 State Key Laboratory of Integrated Optoelectronics, Key Laboratory of UV-Emitting Materials and Technology of Ministry of Education, Northeast Normal University, Changchun, 130024 China; 2 School of Science, Jilin University of Chemical Technology, Jilin, 132022 China; 3 GPL Photon Lab, State Key Laboratory of Luminescence Science and Technology, Changchun Institute of Optics, Fine Mechanics and Physics, Chinese Academy of Sciences, Changchun, 130033 China; 4 Key Laboratory of Functional Materials Physics and Chemistry of Ministry of Education, Jilin Normal University, Changchun, 130103 China; 5 State Key Laboratory of Integrated Optoelectronics, JLU Region, College of Electronic Science and Engineering, Jilin University, Changchun, 130012 China

**Keywords:** Laser material processing, Electronic properties and devices

## Abstract

The integration of surface-regular micro/nanostructured electrodes within a limited footprint area is promising to enhance the electrochemical performance of planar micro-supercapacitors (P-MSCs), while developing simple yet efficient manufacturing methods for such electrodes remains a challenge. Here, we propose a universal strategy combining femtosecond laser plasma lithography with spatial light modulation (SLM-FPL), fabricating well-ordered sub-wavelength micro/nanostructured electrodes of interdigital P-MSCs (SEP-MSCs) on graphene oxide (GO) films. Achieving 500/50 µm finger widths/spacings and 680 nm internal grating periods, this method enables device densities >25 units inch^−2^ with processing efficiency orders of magnitude higher than conventional laser direct writing. Further performance optimizations via wettability modification, electric field engineering, and hybrid composites (GO-MXene/COF) yield outstanding specific capacitance (~41.4 F cm^−3^) and cycling stability (93% retention over 5000 cycles), supporting applications in flexible sensors and compact power supplies. This SLM-FPL technology shows strong potential for high-performance, spatially efficient SEP-MSCs in next-generation integrated systems.

## Abstract (web summary)

Universal femtosecond laser and spatial light modulation enable rapid fabrication of graphene-hybrid micro-supercapacitors on silicon. Simultaneous formation of ordered subwavelength electrode micro/nanostructures boosts processing efficiency and device performance.

## Graphical abstract

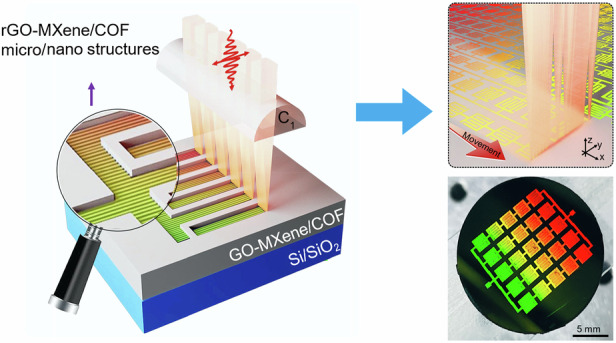

## Introduction

The relentless drive toward characteristic scale miniaturization and heterogeneous integration in micro/nano-optoelectronic devices has necessitated breakthroughs in energy storage technology^1–4^. Developing next-generation portable storage systems, combining high power density and energy density, is critical to overcoming the technology bottleneck^5^. Recently, planar micro-supercapacitors (P-MSCs) stand out as promising electrochemical components among emerging solutions, which offer exceptional power performance, ultra-long cycle life, and rapid charge/discharge capabilities^6,7^. These attributes render them ideal for micro-energy storage, smart electronics, flexible wearable applications, and so on. The electrochemical performance of P-MSCs hinges on both the intrinsic properties of electrode materials and the interfacial structural design^8^. Strategic surface micro/nanostructuring not only amplifies electrode-specific surface areas to expose abundant active sites, but also simultaneously refines charge/ion transport pathways at electrode interfaces^9,10^. Structural optimization enhances the transport efficiency of charges and ions, reduces the internal resistance and diffusion impedance of the electrode, thereby directly enhancing device performance.

Conventional surface micro/nanostructuring includes photolithography, ion beam etching, nanoimprinting, and self-assembly, etc.^11^. In contrast, laser processing has emerged as an alternative, offering non-contact, adaptable operation that combines traditional techniques in precision and versatility^12,13^. In particular, the femtosecond laser processing exemplifies this progress. It combines the foundational benefits of laser-material interactions with unique ultrafast dynamics, enabling sub-wavelength precision, large-area patterning, and deterministic control over micro/nanostructure formation^14–16^. Many emerging technologies based on femtosecond laser processing achieve a critical balance between processing accuracy and throughput while maintaining the flexibility essential for complex 3D architectures at scales below the optical diffraction limit^17–19^. Such advancements position femtosecond laser processing as a competitive frontrunner in energy device manufacturing, where hierarchical structural control and scalability are paramount.

In view of this, here we developed a versatile strategy combined femtosecond laser plasma lithography with spatial light modulation (SLM-FPL) enabling high-efficiency fabrication of silicon-based P-MSC with integrated micro/nanostructured electrodes (SEP-MSC)^20–22^. We first focused the incident femtosecond laser into a line profile on the target graphene oxide (GO) film, then employed spatial light modulator to sculpt the laser’s amplitude distribution. Benefiting from the nonlinear light-matter interaction and photo-reduction capabilities inherent to FPL technology, the relative motion between the focal spot and the GO film led to the formation of reduced graphene oxide (rGO) electrodes, simultaneously patterned with large-area, well-ordered, periodic sub-wavelength micro/nanograting structures in the reduced regions^23^. Laser parameter tuning, including wavelength, power, and scanning speed, enabled precise modulation of electrode surface morphology and GO reduction degree. Remarkably, under a scanning speed of 0.1 mm s^−1^, micro/nanograting was performed on the interdigital electrode region with a footprint of 0.5 × 0.5 cm^2^ of a standard device, requiring ~50 s of processing time. This resulted in the formation of about 7350 laser-induced periodic surface structures (LIPSSs) with spatial period of ~680 nm, demonstrating exceptionally high patterning density and subwavelength-scale fabrication accuracy. That means, under identical laser parameters, this strategy delivers a processing efficiency exceeding 7000-fold compared to conventional direct writing methods. Subsequent wettability measurements and numerical simulations revealed that the structures not only significantly improved surface charge transport properties but also locally enhanced the electric field intensity, thereby contributing to enhanced device performance. The volumetric capacitance of the optimized MSCs was increased by ~4.3 times (~23.7 F cm^−3^) compared to the unstructured devices. To verify universality of the processing technology, similar experiments were further conducted on GO-MXene and GO-covalent organic framework (COF) composite films, where material hybridization enhanced the capacitive behavior of the electrodes, leading to an additional ~2-fold improvement in volumetric capacitance^24,25^. The resulting devices exhibited excellent energy density (2.81 mWh cm^−3^) and power density (~0.32 W cm^−3^). The processing stability and regular surface micro/nanostructures enable exceptional performance uniformity and operational stability, retaining >93% electrochemical performance after 5000 charge-discharge cycles. These performance, coupled with applicability in powering compact circuits and miniaturized sensor systems, underscore the technology’s potential for scalable manufacturing of high-performance SEP-MSC. The work offers further validation for the credibility of the proposed theoretical model concerning FPL processing, along with demonstrating the widespread applicability of the technology^20,23^. Specifically, by emphasizing enhancements in capacitor performance and prioritizing high-quality fabrication, the work transcends mere technological accumulation.

## Results

### Ultrafast Fabrication of SEP-MSC with SLM-FPL technology

**Fig. 1:**
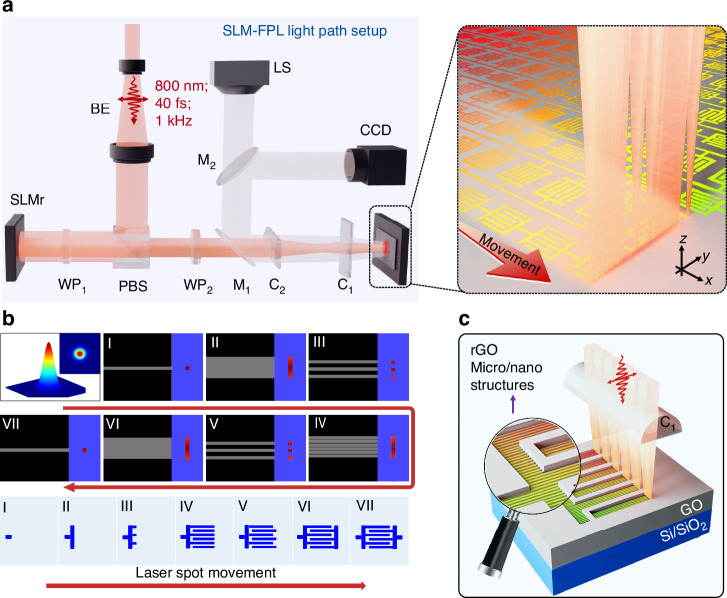
Schematic of the rapid fabrication of arrayed graphene-based SEP-MSCs via SLM-FPL technology. **a** Schematic optical path configuration of the SLM-FPL processing system (left) and the in-situ fabrication of arrayed devices (right). BE, PBS, WP, M, C and LS are the abbreviations for the beam expander, polarizing beam splitter, half-wave plate, mirror, cylindrical lens and light source, respectively. **b** Schematic of the real-time modulation of beam profile enabled by SLMr. **c** Illustration of micro/nanograting structure formation on the electrode surface of SEP-MSCs. The grating orientation is strictly aligned with the polarization direction of incident light

Figure 1a presents a schematic of laser processing setup based on the SLM-FPL technology^20,21^. A femtosecond laser system operates at a central wavelength of 800 nm, with a repetition rate of 1 kHz and a pulse duration of 40 fs. The laser beam with a vertical linear polarization first passes through an expander and polarizing beam splitter (PBS) to uniformly irradiate the aperture of a spatial light modulator (SLMr). Along the reflected beam path, a half-wave plate (WP_1_) rotates the polarization to horizontal for propagation toward the fabrication plane. A second half-wave plate (WP_2_) is then employed to precisely adjust the laser polarization state^20,23^. Concurrently, a pair of orthogonally arranged cylindrical lenses (CL_1_ and CL_2_) performs beam shaping by compressing the Gaussian profile into a linear distribution. The spatially modulated laser beam is then uniformly focused onto a silicon-supported GO film. With synchronized stage movement, large-area, arrayed graphene SEP-MSCs in series or parallel configurations can be fabricated in situ (Fig. 1a right). Following parameter optimization, here the fabrication achieves a 0.1 mm s^−1^ processing speed within a limited 0.5 × 0.5 cm^2^ footprint area of a standard device with interdigital electrodes featuring periodic grating structures of 680 nm. That means the configuration enables a 7000-fold enhancement in processing efficiency under identical operational conditions compared to conventional direct writing methods. For details, see the Materials and Methods section and Supplementary Information SI S1. The entire processing is monitored in real time through a coaxial charge-coupled device (CCD) imaging system.

Notably, the fabrication of above graphene-based SEP-MSC device relies on the synergistic integration of multiple physical mechanisms. First, the photothermal and photochemical effects drive the reduction of GO to its reduced counterparts in laser-irradiated regions. The effective removal of oxygen-containing functional groups (OFGs) creates conductive electrodes in the device, while the unirradiated areas act as charge-blocking separators due to their insulating nature^26^. Second, spatial beam shaping is achieved through dynamic modulation of a commercial SLMr. By precisely controlling the voltage of individual liquid crystal pixels, the grayscale reflection profile of the beam can be dynamically modulated, enabling real-time spatial amplitude shaping of the reflected laser and defining the electrode pattern in the SEP-MSC (see Fig. 1b and Methods)^21^. Finally, the ultrashort pulse duration of the femtosecond laser enables strong nonlinear optical interactions. The laser excites surface plasmon polaritons (SPPs) that interfere with the incident wave, transforming homogeneous irradiation into periodic intensity modulations (Fig. 1c). This creates sub-wavelength grating structures with tunable orientation, periodicity, and aspect ratio, controlled by laser parameters such as polarization, wavelength, power, and repetition rate^20,27^. The cumulative effect of these processes enables efficient, controllable, and rapid fabrication of micro/nanostructured graphene-based SEP-MSC devices. Detailed processing visualization is available in Supplementary Movies S1–4 and the Methods section.

### Surface structuring and photo-reduction of GO films

**Fig. 2:**
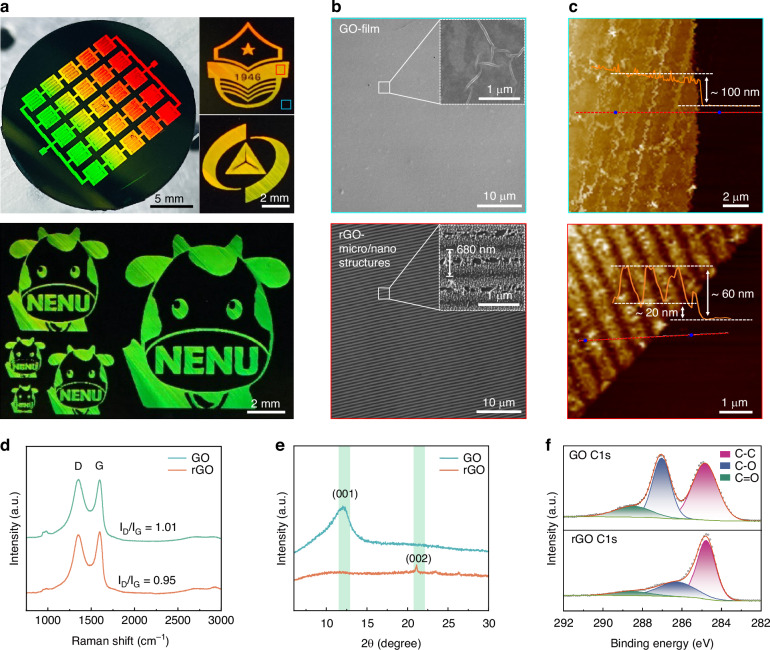
Morphology and characteristics comparison of GO films before and after laser processing. **a** Optical images of the arrayed SEP-MSC devices, university emblems, and “NENU” logos. The regular surface structure makes sample structural colors vary with viewing angles. **b** SEM and **c** AFM images of the GO film before (cyan boxs) and after (red boxs) processing. **d**–**f** Raman, XRD, and XPS spectra of the GO film before and after processing

We first spin-coated the GO dispersion solution onto the surface of a commercial silicon wafer, and achieved precise control over the thickness and uniformity of the film by adjusting parameters such as the number of solution droplet applications, spin-coating speed, and duration (See the Methods section and Supplementary Fig. S2 for details). Subsequently, using the aforementioned SLM-FPL technology, we performed the in-situ synchronous surface micro/nanostructuring and photo-reduction of the silicon-based GO film. Thanks to the editable regulation of the spatial amplitude distribution of the incident light spot by SLMr, the patterned processing on the GO surface exhibited high flexibility. Figure 2a presents the arrayed series-connected SEP-MSCs fabricated by this method, as well as representative badges such as the university emblem and the “cow” logos with different sizes. It is worth noting that due to the existence of periodic micro/nanograting structures on the surface of the processed regions, the optical diffraction phenomenon is obvious and highly dependent on the observation angle. The uniform structural colors macroscopically exhibited by these patterns also reflect the high regularity, periodicity and consistency of the processed surface micro/nanostructures^28^. To further validate the formation mechanism of the structures, comparative morphological analysis was performed on the untreated and laser-processed regions using scanning electron microscopy (SEM) and atomic force microscopy (AFM). Untreated areas exhibited smooth, uniform surfaces with consistent material thickness (~100 nm). In contrast, laser-irradiated regions displayed well-defined periodic micro/nanograting structures characterized by ~680 nm periodicity, duty ratio of ~2:1, and etching depth of ~40 nm (Fig. 2b, c). The overall film thickness also decreased by ~40 nm post-processing. More details about the laser processing can be seen in Supplementary Fig. S3.

The observed reduction in film thickness commonly correlates with the photothermal removal of OFGs during laser-induced rGO formation^20,29^. To validate the mechanism, we comprehensively characterized the chemical composition and crystal structure of the samples before and after laser processing by using Raman spectroscopy, X-ray diffraction (XRD), and X-ray photoelectron spectroscopy (XPS). Raman analysis revealed a slight decrease in the D-to-G peak intensity ratio (I_D_/I_G_) from 1.01 to 0.95 post-processing (Fig. 2d), indicating partial restoration of sp^2^ C-C bonds and an increased degree of graphitization^30^. This change is a typical signature of the conversion from GO to rGO under laser irradiation. Large-scale Raman mapping scans demonstrated that the relative standard deviation (RSD) of these ratio values remained below 1.3%, underscoring the stability and uniformity of the photo-reduction (Supplementary Fig. S4). Further XRD analysis showed significant attenuation of the characteristic GO diffraction peak at 2θ ≈ 10.4°, which corresponds to its highly oxidized layered structure^31^. Another new diffraction peak at 2θ ≈ 21° emerged, confirming more ordered rGO formations after laser processing (Fig. 2e)^32^. Moreover, the reduction behavior is also strongly supported by XPS analysis. In the high-resolution C 1s spectrum of the untreated GO film, characteristic peaks corresponding to C-C, C-O, and C=O bonds were observed at 284.8 eV, 287.0 eV, and 288.4 eV, respectively, with relative contents of 49.3%, 36.8%, and 13.8%. After irradiation, the relative contents of C-O and C=O functional groups decreased to 30.1% and 8.6%, respectively, indicating a significant reduction in OFGs (Fig. 2f)^33^. For more details on the XPS analysis of the GO films before and after laser processing, please refer to the Supplementary Fig. S4.

### Structured electrodes influence on the electrochemical performance of SEP-MSCs

**Fig. 3:**
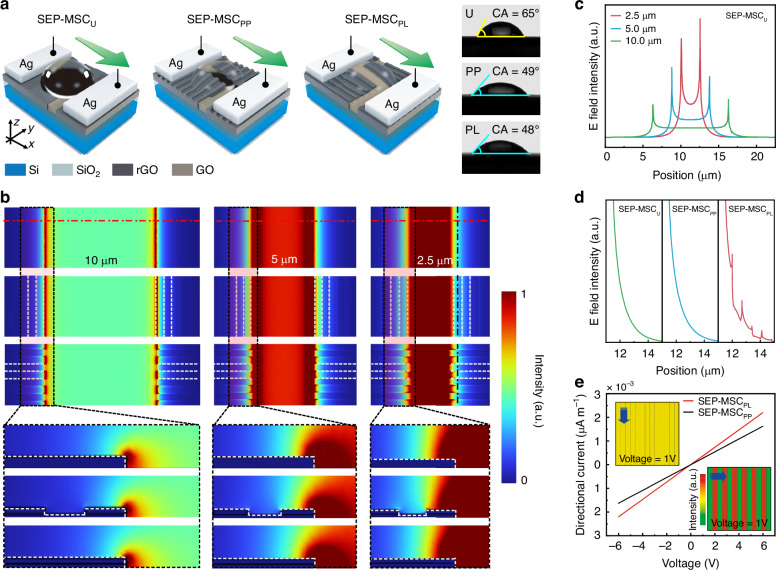
Interfacial wettability and electric field response characteristics under the regulation of electrode surface structures. **a** Comparison of contact angles of rGO films before and after surface structuring. The green arrow represents the direction of the driving electric field. **b** Top and side views of the simulated spatial electric field intensity distribution of films with and without structures, with different channel widths. **c**, **d** Comparison of electric field intensity values extracted along the dashed lines in (**b**). **e** Conductivities comparison of films along and perpendicular to the grating direction

The SLM-FPL technology mentioned above was subsequently employed for the in-situ rapid fabrication of interdigital rGO-based SEP-MSCs. Notably, the micro/nanograting structures introduced onto the electrode surface play a pivotal role in enhancing device electrochemical performance. On one hand, the structures significantly increased the electrode’s specific surface area, thereby providing more electrochemically active sites that improve charge storage efficiency^9,10^. The uniformity of structural dimensions ensured the stability of device performance. On the other hand, the laser-reduced rGO retained partial hydrophilic functional groups (e.g., -OH and -COOH), which could form stable hydrogen bonds with water molecules, endowing the material with excellent wettability^34^. The laser-processed composite structure’s micrometer-scale surface features also enhance hydrophilicity through capillary action^35^. To verify this, contact angle measurements were performed using deionized water dropped onto the structured and unstructured rGO films (Fig. 3a). The unstructured rGO film exhibited a contact angle of ~65°, while the rGO film with structures showed a reduced angle, decreasing by ~16°, indicating improved wettability. This enhanced surface hydrophilicity facilitates closer electrode-electrolyte contact in SEP-MSCs, promoting efficient ion transport within electrode channels, increasing active site utilization, and ultimately improving rate performance^9,10^. Detailed wettability analysis of rGO films and their hybrids is provided in Supplementary Fig. S5.

Moreover, to investigate the impact of surface structuring on electrochemical performance, finite element method (FEM) simulations were conducted to analyze spatial electric field distributions in comparative devices with three different electrode configurations: unstructured device with smooth electrodes (SEP-MSC_U_), device with grating-structured electrodes aligned parallel to the external electric field (SEP-MSC_PL_), and devices with gratings perpendicular to the field (SEP-MSC_PP_) (Fig. 3b, and Methods)^36^. Among the different devices, the spacing between the electrodes was set to 2.5 μm, 5 μm, and 10 μm, respectively. The subsequent simulations revealed two critical phenomena. First, regardless of surface structuring, significant electric field enhancement occurred at electrode edges, with grating structures further amplifying this edge-field intensity, which shows a direct correlate of improved electrochemical performance (Fig. 3b, c). Second, electrode spacing exhibited an inverse relationship with edge-field strength. The shorter inter-electrode distances yielded stronger localized electric fields (Fig. 3b–d, Supplementary Fig. S6). These findings demonstrate that the surface micro/nanostructures can modulate electric field distributions, enhancing charge accumulation and ion transport at the electrode-electrolyte interface^23,36^. Furthermore, the grating structures inherently confer conductive anisotropy to the rGO material, with charge transport properties exhibiting directional dependence^20,23^. When charge carriers propagate parallel to the grating orientation, surface defects exert minimal interference on conductive channel conductivity, yielding superior conduction performance. Conversely, vertical charge transport suffers from interfacial scattering and structural discontinuities that suppress conductivity. The Finite element simulations quantitatively demonstrate this anisotropy under an applied voltage. The parallel-direction integrated volumetric charge density exceeds the vertical counterparts by ~35%, yielding a ratio of 1.35, primarily due to enhanced boundary scattering and energy barrier effects in the perpendicular orientation that disrupt continuous charge pathways (Fig. 3e). The anisotropic behavior closely matches experimental observations, and the directional conductivity disparity directly impacts the electrode-electrolyte interaction (Supplementary Fig. S7). Parallel alignment between grating structures and charge transport paths maximizes electrode-electrolyte interface contact area, shortens ionic adsorption/diffusion pathways, and reduces interfacial resistance, thus favoring overall device electrochemical performance. In contrast, perpendicular orientation degrades interface compatibility, impedes ion migration, and compromises device functionality^36^.

It bears noting that while above studies have demonstrated that laser-treated devices exhibit enhanced performance in terms of material wettability, local electric field enhancement, and anisotropic carrier transport, establishing a rigorous quantitative relationship between these factors with device performance proves challenging. This difficulty arises because the performance improvement stems from the synergistic interplay of multiple factors. Experimentally, when we adjust a specific parameter via laser control, other material properties also undergo alterations. Nonetheless, these finding have laid a theoretical framework for optimizing MSC performance through structured electrode design and have illuminated the path toward precisely modulating interface electric fields and ion transport dynamics.

### Electrochemical performance of rGO-based SEP-MSCs

**Fig. 4:**
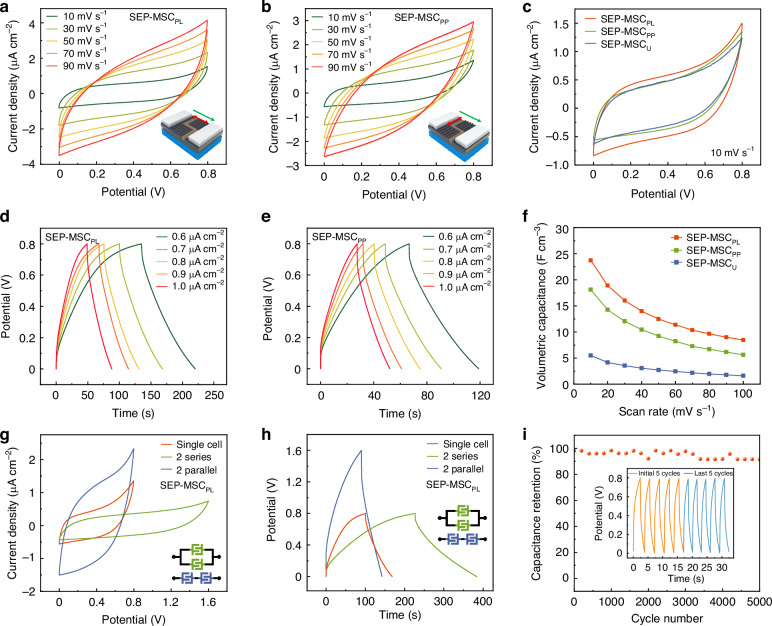
Electrochemical performance comparison among SEP-MSC_U_, SEP-MSC_PL_, and SEP-MSC_PP_ devices. **a**, **b** CV curves of SEP-MSC_PL_ and SEP-MSC_PP_ at different scan rates. The red and green arrows in inserts represent the direction of grating and applied electric field, respectively. **c** Comparative CV curves of SEP-MSC_U_, SEP-MSC_PL_, and SEP-MSC_PP_ at various scan rates. **d**, **e** GCD curves of SEP-MSC_PL_ and SEP-MSC_PP_ under different current densities. **f** Volume-specific capacitance comparison of SEP-MSC_U_, SEP-MSC_PL_, and SEP-MSC_PP_ at varying scan rates. **g**, **h** CV and GCD performance of SEP-MSC_PL_, respectively, in series and parallel configurations. **i** Electrochemical stability measurements of SEP-MSC_PL_ performed at a current density of 3.64 μA cm^−2^

Following the mechanistic elucidation of electrodes with structures impact on the device electrochemical performance, we then fabricated the SEP-MSC_U_, SEP-MSC_PL_, SEP-MSC_PP_ with identical channel spacing of 50 μm and evaluated the performance experimentally. Figure 4a, b shows the cyclic voltammetry (CV) curves of SEP-MSC_PL_ and SEP-MSC_PP_ devices across scan rates from 10 to 90 mV s^−1^, while Fig. 4c compares all the devices at 10 mV s^−1^. All devices exhibited typical rectangular curves characteristic of electric double-layer capacitance, but SEP-MSC_PL_ demonstrated superior performance with the largest enclosed CV area and highest current response, indicating optimal capacitance storage capacity^37^. The CV and galvanostatic charge-discharge (GCD) curve of the contrastive SEP-MSC_U_ is provided in Supplementary Fig. S8. Furthermore, GCD measurements also confirmed these findings through symmetrical discharge profiles (Fig. 4d, e). Notably, SEP-MSC_PL_ maintained performance superiority across multiple testing parameters. Quantitative analysis of volumetric specific capacitance derived from CV data in Fig. 4f revealed that SEP-MSC_PL_ consistently achieved the highest capacitance values. At a scan rate of 10 mV s^−1^, SEP-MSC_PL_ achieves a volumetric capacitance of 23.7 F cm^−3^, which is 4.3 times higher than that of SEP-MSC_U_. This aligns well with our previous analysis showing that micro/nanograting-induced electric field modulation at the electrode interface enhances ion transport efficiency.

Building on the processing versatility of SLM-FPL technology, we further developed the integrated device arrays using SEP-MSC_PL_ units with superior performance as building blocks and conducted systematic electrochemical characterization. Two representative series and parallel configurations were selected for detailed analysis. Electrochemical measurements revealed that the parallel integration significantly enhanced the total capacitance, while the series arrangement effectively expanded the operating voltage window of the device ensemble (Fig. 4g, h). Notably, all the configurations maintained stable during prolonged cycling. A single SEP-MSC_PL_ unit demonstrated exceptional durability, retaining over 93% of its initial capacitance after 5000 galvanostatic charge-discharge cycles (Fig. 4i). These findings validate the technological compatibility and performance reliability of SLM-FPL in fabricating SEP-MSC devices, offering a practical and scalable route toward highly integrated and durable on-chip energy storage units.

### Performance optimization and application of GO-composite-based SEP-MSCs

**Fig. 5:**
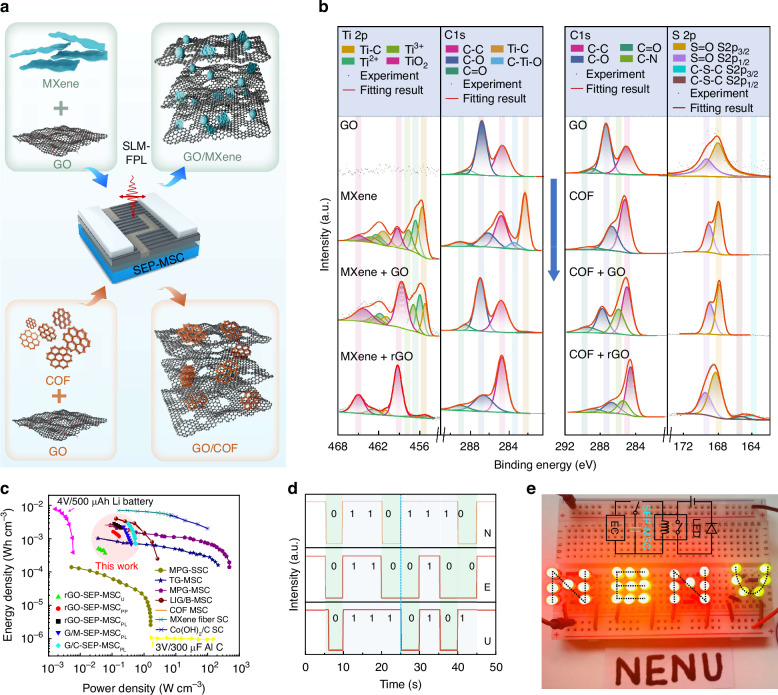
Performance analysis and application demonstration of the SEP-MCSs based on graphene hybrids. Schematic (**a**) and XPS spectra (**b**) comparison of composite component changes of SEP-MCS before and after laser processing. **c** Performance comparison of our devices with the reported MSCs^42–47^. The G/M-SEP-MSC_PL_, G/C-SEP-MSC_PL_ and rGO-SEP-MSC_PL_ represent the SEP-MSC_PL_ devices made of rGO/MXene, rGO/COF and rGO, respectively. Demonstration of COF/rGO SEP-MCS_PL_ powering a pressure sensors (**d**) and a voltage comparator circuit (**e**)

To further optimize the electrochemical performance of our SEP-MSC devices, we developed composite electrodes by integrating GO respectively with advanced inorganic Ti_3_C_2_T_x_ MXene and covalent organic framework (COF) nanosheets, both renowned for their exceptional capacitive properties^24,25^. These hybrid materials were also patterned into interdigitated SEP-MSC_PL_ devices via the SLM-FPL technology (Fig. 5a and Supplementary Fig. S9). Periodic micro/nanograting structures with well-defined orders were successfully fabricated on composite electrodes. The 2D fast Fourier transform (2D-FFT) spectra show the periods are respectively 680 ± 6.8 nm and 680 ± 4.5 nm, indicating good uniformity of gratings over a large area. Then, XPS characterization was used to reveal the structural transformations induced by laser processing. For the GO-MXene composite, post-laser treatment significantly reduced Ti-C bonds, Ti^3+^/Ti^2+^ species, and oxygen-containing C-O/C=O groups, while concurrently enhancing Ti-O bonds (2p_3/2_/2p_1/2_, ~459.3/461.1 eV) and C-C components. This transformation indicates the formation of a carbon-dominated hybrid conductive network with oxidized TiO_2_ phases, which introduces abundant pseudocapacitive active sites (Fig. 5b left)^38,39^. In contrast, GO-COF composites retained characteristic COF features post-processing, including C-N (~285.6 eV), S=O (~168.42/169.6 eV), and C-S-C (~164.2/165.44 eV) bonds, while increasing C-C proportions (Fig. 5b right). This suggests laser-induced formation of a carbon-based hybrid nano-skeletal framework interconnected with original COF moieties, where COF-derived nitrogen and sulfur atoms introduce additional electrochemical active sites and enhance charge transfer kinetics (Supplementary Fig. S10)^40,41^. The synergistic effects of these material optimizations and structured electrode design yielded substantial performance improvements, achieving a volumetric capacitance of ~41.4 F cm^−3^ in CV value. Ragone plot analysis (Fig. 5c) demonstrated that our device outperforms many reported MSCs, delivering an energy density of ~2.81 mWh cm^−3^ while maintaining power density of ~0.32 W cm^−3^ (Supplementary Fig. S11)^42–47^.

To validate the applicability of our SEP-MSCs, we designed and showcased two representative use cases. In the first scenario, a pre-charged SEP-MSC unit via electrochemical workstation was integrated with a flexible pressure sensor to establish a self-powered mechanical sensing system^48^. The device supplied operating voltage to the sensor, where real-time pressure changes were reflected through output voltage modulation. Specifically, mechanical stimulation triggered a voltage defined as zero, while pressure release restored the output to the device’s open-circuit voltage. To simplify data processing and improve signal recognition efficiency, the sensor’s output voltage underwent normalization before analysis. Figure 5d shows the binary encoding-based pattern recognition results of the letters “N”, “E” and “U” based on the mechanism, visually demonstrating the potential for flexible human-machine interfaces and information recognition systems. In the second demonstration, the SEP-MSC was employed as an independent micro-power source to drive a voltage comparator circuit, testing its capability in logic control applications^49^. A test system was constructed based on the classic working model (Fig. 5e). Following stable charging, the device’s output was connected to the comparator’s input. When the device output reached the preset threshold voltage of 0.3 V, the comparator was activated, immediately illuminating an output-connected light-emitting diode. This rapid response confirmed the ability of our SEP-MSC device to reliably trigger low-power electronic components. A video of the experiments is provided in Supplementary Movie S5.

The above results comprehensively demonstrate the practical viability of our SEP-MSC devices for power supplies in portable electronics, flexible sensor networks, and microscale logic systems. They highlight the distinctive capabilities of our SLM-FPL technology in enabling precise fabrication and processing of SEP-MSC devices on silicon substrates, which holds significant promise for advancing on-chip intelligent sensing applications. However, current fabrication methodologies exhibit certain limitations in device manufacturing efficiency, flexibility of electrode surface micro/nanostructuring, and overall capacitance performance. These challenges present opportunities for improvement through systematic optimization. Notably, the inherent strengths of SLM-FPL technology, particularly its capacity for microscopic nonlinear light-matter interactions and macroscopic optical path control, provide a robust foundation for addressing these limitations. By refining laser processing parameters and device structural configurations, we anticipate breakthroughs in fabrication efficiency and structural adaptability. For example, emerging researches have indicated that the nonlinear mechanisms inherent to FPL technology could enable the creation of deep sub-wavelength periodic structures on 2D film surfaces^50^. When combined with optimized optical path design and post-processing techniques, this capability may facilitate the fabrication of more complex multi-layer architectures^51^. For SEP-MSC devices, performance enhancements could also be achieved through material-level innovations, including increased energy storage material thickness, optimized composite systems, and hierarchical electrode structures^52^. Such advancements would not only elevate electrochemical performance but also broaden application scope, solidifying the technological foundation for integrated micro-power solutions in advanced sensing and portable electronics. Looking ahead, through the optimization of experimental parameters, such as augmenting laser power and scaling up the dimensions of optical components, and integrating it with complementary linkage equipment, the SLM-FPL technology is poised to transition into practical applications especially the on-chip energy systems.

## Discussion

In conclusion, we have demonstrated a versatile and high-efficiency strategy for fabricating SEP-MSCs by synergizing SLM-FPL technology. Leveraging the nonlinear light-material interactions inherent to femtosecond lasers and the programmable patterning flexibility of SLMr, the method enables large-area arrayed interdigital devices fabrication on diverse materials while achieving in-situ regular sub-wavelength micro/nanograting structuring with period of ~680 nm within confined electrode regions. Multiple mechanisms, such as increasing electrode-specific surface area via hierarchical structures, optimizing ion transportation through wettability modulation and localized electric field engineering, and synergistic properties from hybrid composites (GO, GO-MXene and GO-COF), have been considered during processing to enhance the device electrochemical performances. A representative implementation demonstrates exceeding 25 devices per square inch with outstanding energy density (2.81 mWh cm^−3^), power density (~0.32 W cm^−3^) and over 93% capacitance retention after 5000 cycles, validating its practical viability for flexible sensing systems and compact power modules. Our SLM-FPL technology provides a feasible platform for tailoring surface structuring functionalities and optimizing energy storage interfaces, presenting technical compatibility for the development of next-generation miniaturized energy storage devices with high integration density and reliability.

## Materials and methods

### Preparation of GO, GO-MXene, and GO-COF

GO dispersion (2 mg mL^−1^, flake size 20–30 μm, Gaoxi Tech Co., Ltd) was drop-cast onto oxygen plasma-pretreated Si/SiO_2_ wafers, followed by repeated casting for controlled thickness and room-temperature drying before laser processing. For MXene/GO films, Ti_3_AlC_2_ MAX phase was synthesized by sintering Ti, Al, and C powders (3:1:2 molar ratio) at 1650 °C under argon for 2 h, then ball-milled, sieved, and etched in 12 M LiF/9 M HCl for 24 h to remove Al. After rinsing to neutral, the product was ultrasonicated under argon in an ice bath for 20 minutes, and the supernatant (Ti_3_C_2_T_x_) was mixed with GO at 1:12 mass ratio for homogeneous suspension before drop-casting. For COF/GO films, Tp (128 mg, 0.61 mmol) and Pa-SO_3_H (172 mg, 0.92 mmol) were dissolved in 10 mL DMSO each, sonicated for 15 min, then the aldehyde solution was dropwise added to the amine solution under stirring. The mixture was kept at 60 °C for 48 h to form a COF, which was blended with GO at 1:10 mass ratio, stirred, and drop-cast following the same procedure. All the reagents and solvents used here were purchased from commercial sources and were used without further purification.

### Femtosecond laser fabrication

The SEP-MSCs with large-area, well-ordered, periodic sub-wavelength micro/nanogratings on electrodes were fabricated using a commercial chirped-pulse amplification femtosecond laser system (Spitfire Ace, Spectra Physics), delivering 800 nm wavelength, 40 fs pulse duration, and a repetition rate of 1 kHz. The laser beam, modulated by a SLMr (Hamamatsu, LCOS-SLM X13138-02, Output wavelength: 800 ± 50 nm; Light utilization efficiency: 97% @ 800 nm; Resolution: 1272 × 1024; Active area: 15.9 × 12.8 mm), was focused into a line-shaped spot on the film sample surface using two cylindrical lenses. The device was fabricated using a laser power maintained at 120 mW and a scanning speed set to 0.1 mm s^−1^. Pattern generation was based on a 256 × 256 pixel grayscale image, enabling grayscale variation along the y-axis of the SLMr microplane. The grayscale values were used to simulate the aperture state, establishing a direct correlation between laser intensity distribution and electrode geometry. The patterning process was controlled in real time using a LabVIEW program, which synchronized SLMr modulation with three-axis stage movement to achieve precise writing of the designed structures.

Notably, the processing efficiency enhancement mentioned above refers to the ratio of fabrication throughput between FPL and conventional direct writing laser technique. Within the standard device area measuring ~0.5 × 0.5 cm^2^, there are ~7350 grooves with a period of 680 nm. The grooves can be generated in a single pass of the light spot by employing FPL, whereas traditional direct writing necessitates multiple reciprocating movements of light spot to accomplish the same task. Although theoretically, enhancing the direct writing speed could boost efficiency, given factors such as sub-wavelength processing accuracy and the fragility of the film material, merely increasing the speed fails to effectively address the issue of processing throughput. More information can be seen in Supplementary Fig. S1.

### Electric field characteristic simulations of the structured electrodes

The electric field characteristics of the electrodes was simulated using COMSOL Multiphysics’ Electrostatics and Electric Currents modules. A realistic MSC geometry was modeled to calculate the global potential and electric field distributions, considering channel widths ranging from 2.5 μm to 250 μm. For analysis of local electric field behavior, a simplified symmetric structure with opposing electrode pairs was developed, incorporating periodic grating micro/nanostructures on the electrode surfaces. Boundary conditions were set with one electrode at 0 V and the opposite electrode at 1 V, matching experimental conditions. The electric field intensity was calculated using the standard formulation.

1 $$\nabla \cdot D=\rho$$

2 $${\boldsymbol{E}}=-\nabla V$$

Where ***D*** is the electric displacement, *ρ* is the space charge density, ***E*** is the electric field, and *V* is the electric potential. Electrical anisotropy of the grating structures was evaluated using COMSOL Multiphysics’ Electric Currents module for steady-state current analysis, focusing on electronic conductivity. The model incorporated periodically arranged conductive/insulating gratings with dimensional parameters matching experimental configurations. Electric field orientations were applied parallel and perpendicular to the grating direction, while other boundaries were assigned electrically insulating conditions. A pronounced conductivity contrast was defined between conductive and insulating regions. The current density vector field (***J***) was extracted, and its component along the electric field direction was volume-integrated to quantify electrical anisotropy.

3 $${I}_{X}={\int }_{\!\!V}{{J}}_{X}{\rm{d}}V$$

### Properties and electrochemical performance characterizations of MSCs

Optical microscopy images were acquired using a confocal laser microscope (Keyence, VK-X1000), while morphological analysis at the nanoscale was achieved via field-emission scanning electron microscopy (FE-SEM, Hitachi, SU8600). Film topography were quantified in tapping mode using an AFM (Bruker, Dimension Icon). Material composition and crystal structure were investigated through confocal Raman spectroscopy (Horiba, LabRAM HR-Evolution) using a 532 nm laser source, XPS (AXIS SUPRA+), and XRD (Rigaku D/max-2500 X-ray diffractometer). The contact angle was measured using contact angle goniometer (POWEREACH, JC2000D3) by dropping 7 μL of deionized water.

Electrochemical performance was evaluated using a CHI 760E electrochemical workstation with silver wires employed for electrode interconnection. A solid-state H_3_PO_4_-PVA gel electrolyte was deposited onto the electrode active regions. The electrolyte was prepared by dissolving 6 g of H_3_PO_4_ in 60 mL deionized water, followed by gradual addition of 6 g PVA powder under continuous stirring. The mixture was heated to 85 °C and maintained under vigorous agitation until a transparent gel formed, then allowed to cool to room temperature under ambient ventilation prior to application.

Based on the CV curves, the areal capacitance (*C*_*A*_, in F cm^−2^) was calculated using the following equation.

4 $${C}_{A}=\frac{1}{S\times v\times \Delta V}{\int }_{{\!\!V}_{i}}^{{V}_{f}}I(V)dV$$

Where *S* is the area of the active electrode (cm^2^), Δ*V* is the voltage window (V), *ν* is the scan rate (V s^−1^), and *V*_*f*_ and *V*_*i*_ are the upper and lower voltage limits of the CV curve, respectively. *I*(*V*) represents the current in amperes at a given voltage, and the integral ${\int }_{{V}_{i}}^{{V}_{f}}I(V)dV$ corresponds to the enclosed area under the CV curve. The *C*_*A*_ at different current densities was further calculated from the GCD curves using the corresponding formula.

5 $${C}_{A}=\frac{1}{S\times (dV/dt)}$$

Where *I* is the discharge current (A), and d*V*/d*t* is the voltage change rate of the constant-current discharge curve. The *C*_*V*_ was calculated using the following formula.

6 $${C}_{V}=\frac{{C}_{A}}{d}$$

Where *d* denotes the effective thickness of the electrode. The volumetric energy density (*E*_*V*_, Wh m^−3^) is calculated using the following equation:

7 $${E}_{V}=\frac{1}{2}\times {C}_{A}\times \frac{{(\Delta V)}^{2}}{3600}$$

Where Δ*V* = *V*_max_ – *V*_drop_ represents the discharge voltage window (V). The *V*_max_ is the maximum voltage and *V*_drop_ is the initial voltage drop at the beginning of the discharge plateau, representing the voltage drop phenomenon. The volumetric power density (*P*_*V*_, W cm^−3^).

8 $${P}_{V}=\frac{{E}_{V}}{\Delta t}\times 3600$$

Where Δ*t* represents the discharge time (S).

## Supplementary information

Supplementary Information for High-efficiency femtosecond laser fabrication of graphene-hybrid planar micro-supercapacitors with micro/nanostructured electrodes

Supplementary movie 1

Supplementary movie 2

Supplementary movie 3

Supplementary movie 4

Supplementary movie 5

## Data Availability

All data are available in the main text or the supplementary materials. Additional data supporting the findings of this study are available from the corresponding author upon reasonable request.
