# Supplementary material for: High-efficiency femtosecond laser fabrication of graphene-hybrid planar micro-supercapacitors with micro/nanostructured electrodes
Source: Light Sci Appl. 2026 Jan 21;15:75. doi: 10.1038/s41377-025-02182-5 (PMC12819388; doi:10.1038/s41377-025-02182-5)
Supplement: Supplementary file 1 — Supplementary Information for High-efficiency femtosecond laser fabrication of graphene-hybrid planar micro-supercapacitors with micro/nanostructured electrodes [file 41377_2025_2182_MOESM1_ESM.docx]

Supplementary Information for

**High-efficiency femtosecond laser fabrication of graphene-hybrid planar micro-****supercapacitors with micro/nanostructured electrodes**

Yuyuan Zhang^1,2†^, Tingting Zou ^3†^, Haobo Jiang^4^, Xiuyan Fu^4^, Wei Xin^1^*, Yiyang Meng^1^, Xilin Li^5^, Jun-Ming Cao^1^, Lin Yang^1^, Yuanzheng Li^1^, Weizhen Liu^1^*, Dongdong Han^5^, Xing-Long Wu^1^, Jianjun Yang^3^, Haiyang Xu^1^* and Yichun Liu^1^

^1^State Key Laboratory of Integrated Optoelectronics, Key Laboratory of UV-Emitting Materials and Technology of Ministry of Education, Northeast Normal University, Changchun 130024, China

^2^School of Science, Jilin University of Chemical Technology, Jilin 132022, China

^3^GPL Photon Lab, State Key Laboratory of Luminescence Science and Technology, Changchun Institute of Optics, Fine Mechanics and Physics, Chinese Academy of Sciences, Changchun 130033, China

^4^Key Laboratory of Functional Materials Physics and Chemistry of Ministry of Education, Jilin Normal University, Changchun 130103, China

^5^State Key Laboratory of Integrated Optoelectronics, JLU Region, College of Electronic Science and Engineering, Jilin University, Changchun 130012, China

†These authors contributed equally to this work.

*Email: xinwei@nenu.edu.cn, wzliu@nenu.edu.cn and hyxu@nenu.edu.cn

SI S1. SEP-MSC device fabrication by using FPL technology


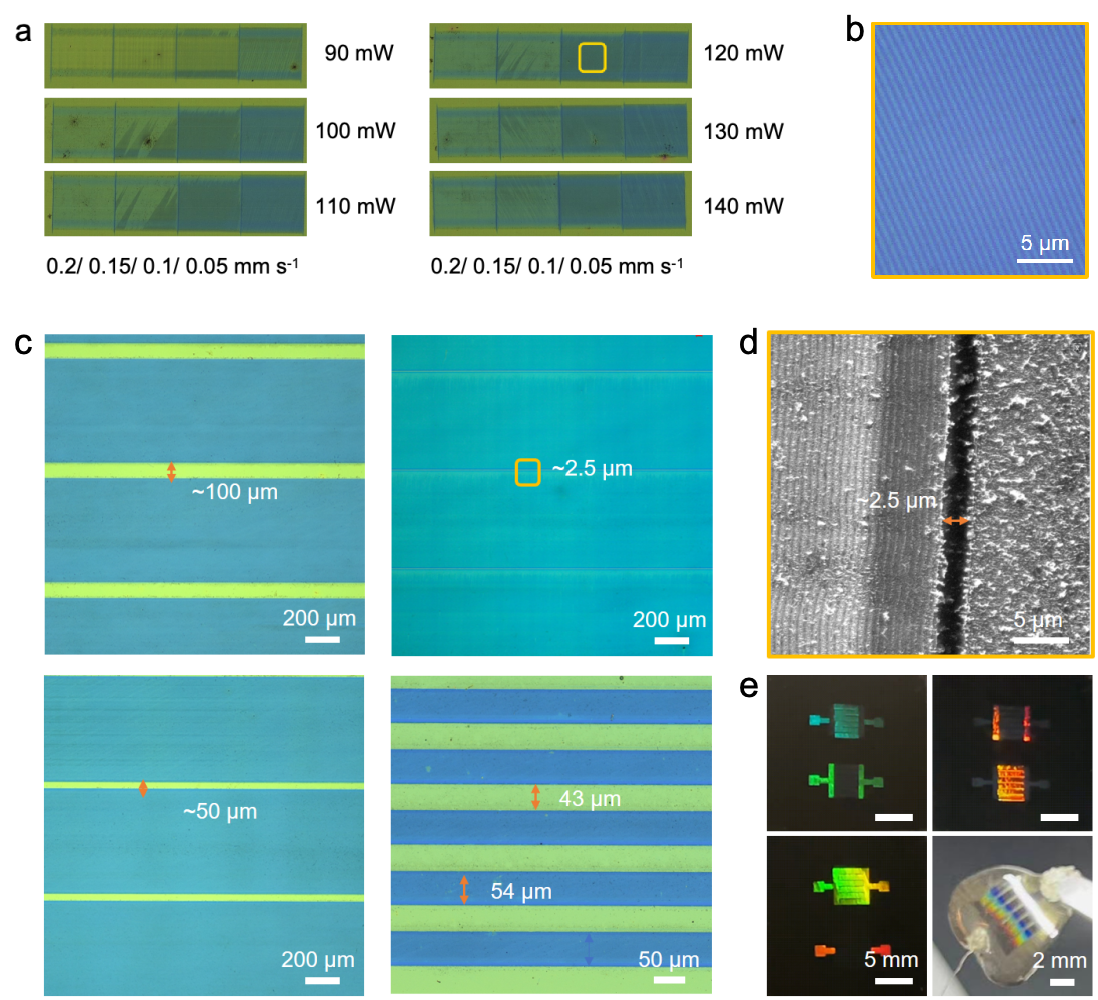


**Figure S1. Morphology of samples processed under different parameters.** a-c) Optical images of rGO LIPSS regions processed under different laser powers and scanning speeds. d) SEM image of the electrode with a 2.5 μm gap. e) Structural color and flexible demonstration of the samples.

Utilizing the FPL laser processing optical path delineated in the main text, the silicon-based SEP-MSC devices on GO films can be fabricated by fine-tuning laser parameters, including the movement speed of the laser spot, and the power density, repetition frequency, polarization state of light. **Figure S1a** illustrates the macroscopic morphology of processed samples under different parameters. Following parameter optimization, large-area, well-ordered, sub-wavelength grating structures can be directly induced within the laser spot’s irradiated region (**Figure S1b**). The orientation of these grooves aligns parallel to the laser’s polarization direction, and altering the incident light’s polarization state can correspondingly adjust the groove direction. Additionally, integrating spatial light modulation (SLM) technology enables direct processing of SEP-MSCs with interdigital electrodes on the film surface, with the minimum electrode spacing achievable at 2.5 μm (**Figure S1c-1d**).

Notably, two points deserve further clarification here:

First, as previous report, the induced sub-wavelength gratings based on FPL technique mainly originate from the interference between the incident light field and the excited surface plasmon polaritons (SPPs) during femtosecond laser irradiation.^1^ This process is accompanied by two types of nonlinear light-matter interactions. Upon irradiation, multiphoton absorption and nonlinear ionization first induce a depth-dependent gradient photochemical reduction in the GO film, forming a decreasing dielectric-permittivity distribution from the surface to the interior. This gradient enables the excitation of transverse electric mode SPPs at the air-GO interface. Then, the interference between the incident light and the excited SPP waves produces a periodic energy deposition (period of ~680 nm), which further promotes the localized ablation/photo-reduction and ultimately lead to the formation of uniform, large-area sub-wavelength gratings. Notably, this energy deposition process exhibits nonlinear feedback characteristics.^2^ The positive nonlocal feedback enhances the scattered field and drives the lateral ordering of structures, while the negative feedback such as reaction saturation or energy dissipation suppresses excessive growth and stabilizes the patterns. The ablation threshold of the fabricated material serves as the primary source of nonlinearity, defining the regions where energy deposition and material modification occur. The nonlinear feedback endows the process with strong self-regulation and robustness. This also ensures that we can transfer the fabricated samples to other substrates (such as flexible substrates) while maintaining the quality of the sample’s microstructures, as shown in **Figure S1e**.

Second, the processing efficiency enhancement of FPL technology refers to the ratio of fabrication throughput between FPL and conventional laser direct writing technique. In our experiment, the laser beam was first shaped into a “line-shaped” profile by a cylindrical lens. Owing to the interference mechanism of FPL, the graphene hybrid film within the beam coverage area was simultaneously patterned into gratings with a period of ~680 nm and a duty cycle of about 1:1. Accordingly, for a standard device used here with a footprint of ~0.5×0.5 cm^2^, the total processing time depends primarily on the ratio between the beam travel length and the movement speed of the laser spot. Under identical scanning parameters, the ratio of fabrication throughput between the FPL and laser direct writing processes can be approximated by the width ratio of the device (0.5 cm) to the groove period (680 nm). As a result, we derive that the fabrication efficiency is improved by about 7000 times. Notably, the defined fabrication efficiency here is obviously related to the footprint area of the electrode region. Theoretically, if the beam size or the device footprint changes, the corresponding fabrication efficiency enhancement value will also vary. However, some factors must be considered simultaneously in experiments, such as the influence of laser power on the grating uniformity/damage extent, or the device footprint on its electrochemical performance. These considerations prevent arbitrary adjustment of the laser parameters. In addition, we defined the fabrication throughput comparison under the condition of an identical laser spot movement speed. It can be expected that if the speed of laser direct writing increased, the efficiency would decrease accordingly. However, the experimental situation is also complex. The graphene film thickness here is only several hundred nanometers, and the groove width is about 340 nm (period of 680 nm). Simply increasing the scanning speed in direct writing to enhance throughput would make it difficult to maintain fabrication structural quality. This conclusion can also be observed from the **Figure S1a**. High-quality structures can be achieved only within a narrow processing window. Therefore, the fabrication efficiency enhancement referred here should be regarded as a conceptual verification obtained under specific experimental conditions, demonstrating the significant potential for throughput improvement of FPL technique in our experiment. The parallel processing method mentioned above, which leverages nonlinear light-matter interactions, exhibits substantial potential for achieving remarkable advancements in both the quality and yield of device fabrication. Nevertheless, constrained by current experimental conditions, this technology remains at the laboratory verification stage. Looking ahead, through the optimization of experimental parameters—such as augmenting laser power and scaling up the dimensions of optical components—and integrating it with complementary linkage equipment, this technology is poised to transition into practical applications.

SI S2. Schematic drop-coating of GO solution for film formation


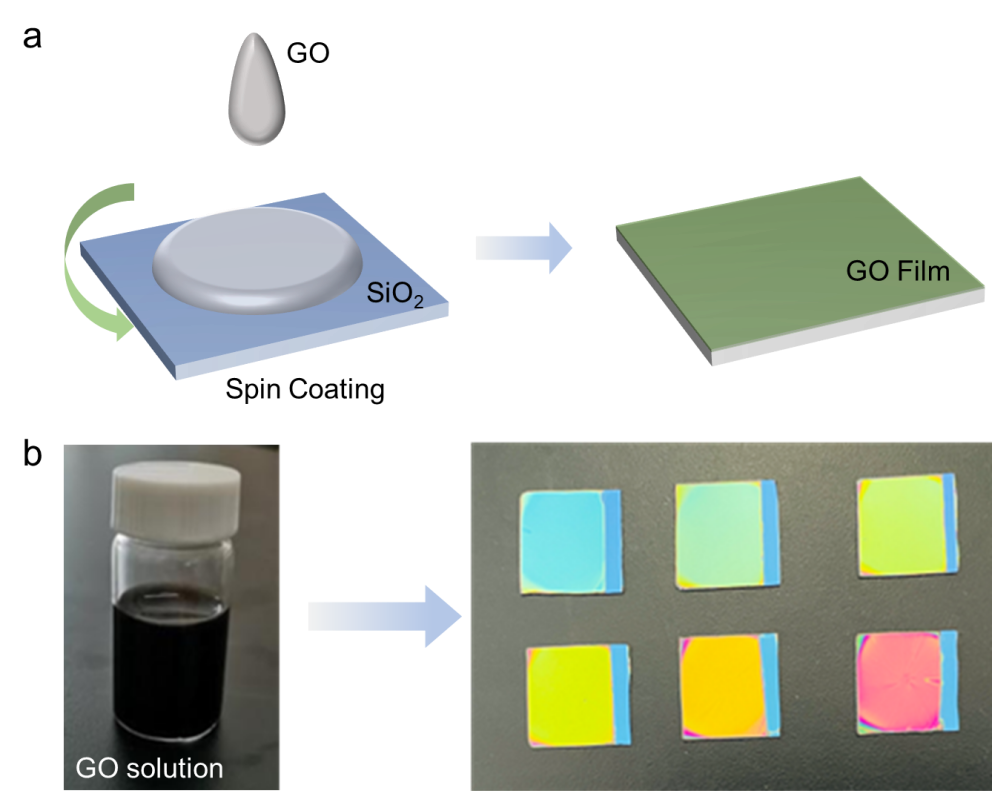


**Figure S2. Schematic drop-coating of GO solution for film formation.** a) Schematic illustration of the drop-coating process for GO film formation. b) Optical images of GO films prepared at 2400 rpm for 30 s with different coating cycles (15–40 times).

A 2 mg mL^-1^ GO solution was used for drop coating at a spinning rate of 2400 rpm. For each coating cycle, the solution was allowed to remain on the substrate for 30 s before spinning. Films were prepared by applying 15, 20, 25, 30, 35, and 40 coating cycles, respectively. Tape was attached along the film edges to assist thickness measurement. The film thickness increases with the number of coating cycles. The film with a thickness of 100 nm was selected as the substrate for subsequent laser processing.

SI S3. Micro-morphology of rGO sample surface after laser processing


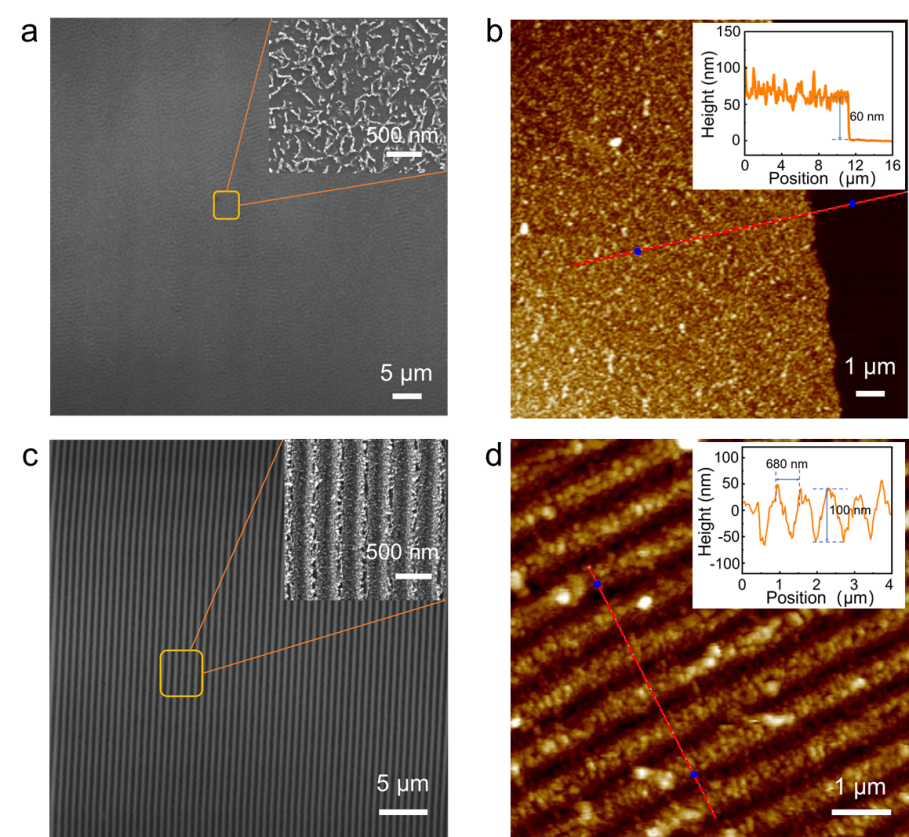


**Figure S3. Micro-morphology of rGO sample surface after laser processing.** a, c) SEM images of rGO samples without and with LIPSS structures. b, d) AFM images of rGO samples without and with LIPSS structures.

To explore laser polarization effects on surface structuring and its implications for electrode functionality, circularly polarized femtosecond pulses (800 nm, 35 fs) were generated via a quarter-wave plate. Circular polarization produced stochastic surface textures with shallow modulation (~60 nm residual thickness), while linear polarization enabled precise fabrication of ordered LIPSS exceeding 100 nm depth. This dual capability demonstrates femtosecond laser processing’s adaptability in creating tailored nano-architectures, offering a versatile platform for optimizing electrode-electrolyte interfaces in energy storage applications.

SI S4. Raman mapping and XPS spectra of GO and rGO samples


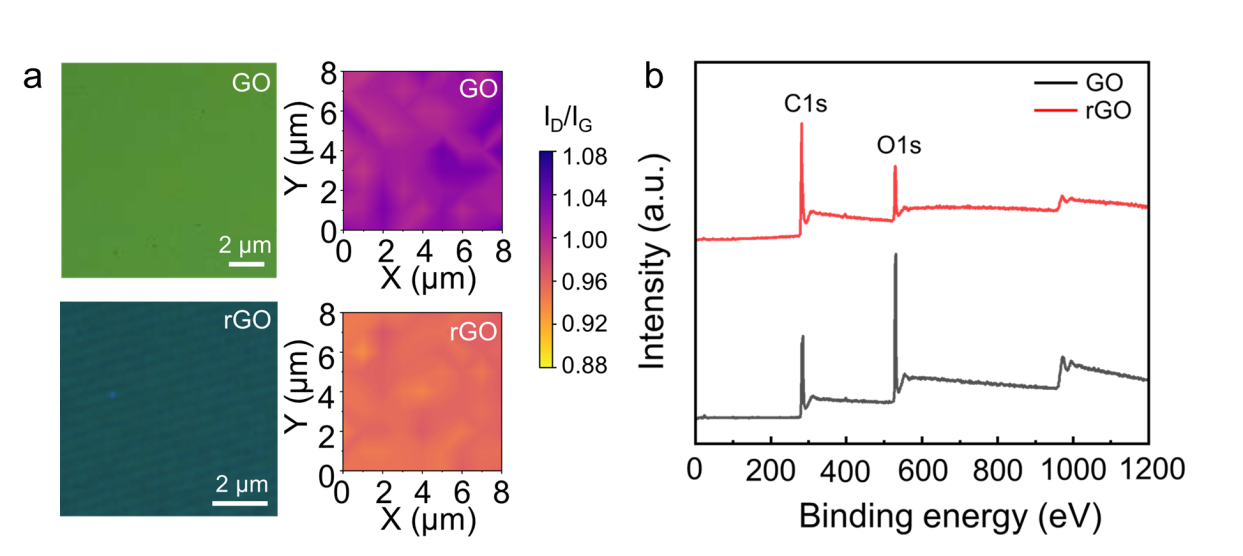


**Figure S4. Raman and XPS characterizations of GO and rGO samples.** a) Raman mapping of GO and rGO showing the ID/IG distributions across the scanned regions. b) XPS survey spectra of GO and rGO samples before and after laser processing.

**Figure S4a** presents the Raman mapping of GO and rGO-LIPSS samples, respectively. The I_D_/I_G_ mapping distributions in the representative regions of GO and rGO samples remain uniform, and the I_D_/I_G_ values of GO are generally higher than that of rGO. This suggests that the sp^2^ carbon network in rGO was partially restored after laser reduction, which confirms the reliability of the observed trend. It is noteworthy that these ratio values primarily indicate an enhanced reductive degree of the material post-laser treatment. Given that the reductive degree is intrinsically linked to the material’s conductivity, and considering the role as an electrode in the device, an improvement in electrode conductivity directly correlates with enhanced charge collection efficiency and overall device performance. Consequently, Raman spectroscopy results can, to a certain extent, directly gauge the device’s performance. Nevertheless, owing to the lack of a direct correlation between the two, conducting a quantitative analysis of their relationship remains challenging.

The XPS spectra of GO and rGO before and after laser processing are shown in **Figure S4b**. A significant decrease in the relative intensity of the O 1s peak is observed after processing, with the oxygen content reduced. This indicates that the laser treatment effectively removed oxygen-containing functional groups from the GO surface, resulting in successful reduction.^3^

SI S5. Effect of micro/nanostructures on the wettability of water and gel electrolytes


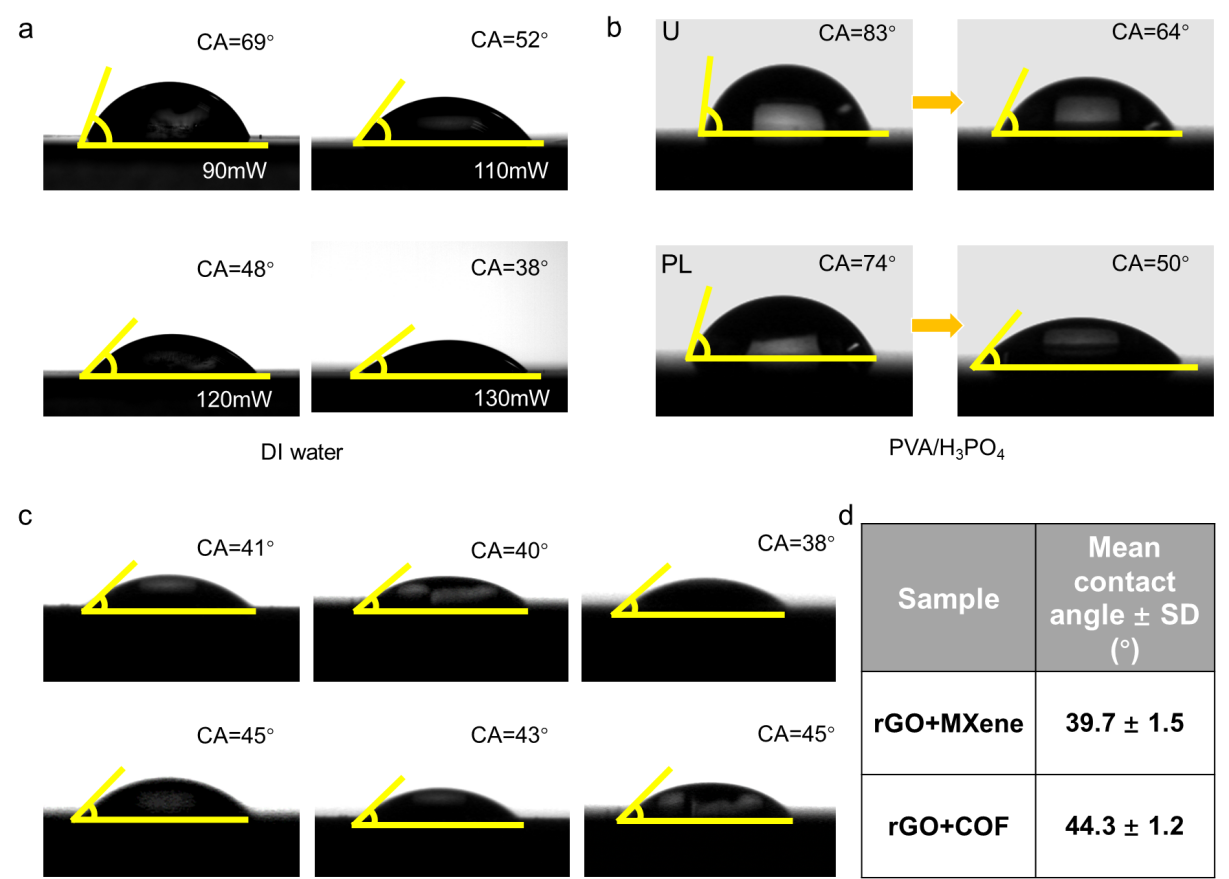


**Figure S5. Effect of micro/nanostructures on the wettability of water and gel electrolytes.** a) Water contact angles on micro/nanostructured surfaces prepared under different laser powers. b) Initial and 2-minute contact angles of the electrolyte on structured and unstructured surfaces at a laser power of 120 mW. c) Deionized water contact angles at different positions on the processed structured surfaces of the rGO/MXene and rGO/COF composite samples.

**Figure S5a** shows that, at a fixed scanning speed of 0.1 mm s^-1^, the water contact angle gradually decreased with increasing laser power, indicating enhanced surface hydrophilicity. **Figure S5b** illustrates the wetting behavior of the PVA-H_3_PO_4_ gel electrolyte on both structured and unstructured electrode surfaces processed at 120 mW. **Figure S5c** presents the water contact angles of the laser-processed rGO/MXene and rGO/COF composite films at different positions. The small relative standard deviations (RSD<3.8%, **Figure S5d**) show consistent wettability across the micro/nanostructured sufaces.

SI S6. Simulated electric field distributions


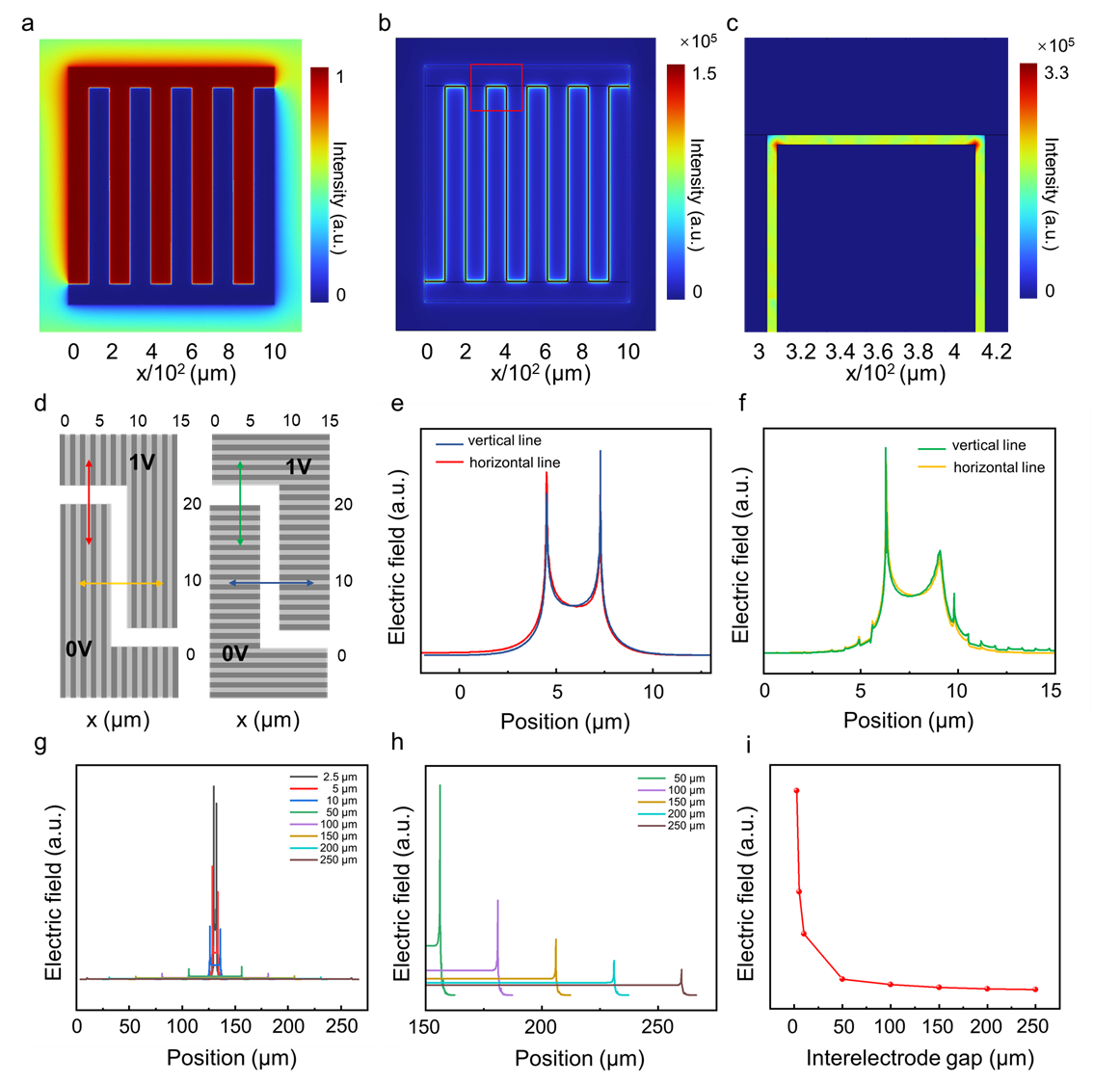


**Figure S6. Simulated electric field distributions.** Simulated electric field distributions of interdigitated electrodes. a-c) Potential and electric field intensity distributions of the interdigitated region. The gap is set as 2.5 μm. d) Models of interdigitated electrodes with horizontal and vertical LIPSS. e-f) Electric field intensities along different paths in Figure S6d. g**-**i) Electric field intensity distributions for interdigitated electrodes with gaps changed from 2.5 μm to 200 μm.

To investigate the electric field characteristics of interdigitated electrodes of our MSCs, we simulated potential and electric field distributions of them with different electrode gaps. The results reveal strong field concentration at electrode edges, particularly at sharp corners.^4^ Further comparison of electric field intensities extracted along different spatial positions—parallel and perpendicular to the grating orientation—shows consistent field enhancement, confirming that periodic micro/nanostructures amplify local electric fields regardless of direction. It should be noted that, for both SEP-MSC_PL_ and SEP-MSC_PP_ devices, the internal micro/nanostructure architectures consistently comprise gratings oriented both parallel and perpendicular to the applied electric field. Additionally, the electric field enhancement at regions far away from the electrode edges becomes progressively less pronounced. Consequently, the orientation of grating structures within the finger electrodes exerts a less pronounced influence on device electrochemical performance, while manifesting predominantly through variations in conductive film conductivity. **Figure S6e** and **6f** illustrate this phenomenon by presenting simulated electric field intensities measured along colored pathways in each sample (**Figure S6d**). Critical observation reveals that within structurally analogous local regions, the electric field intensity distributions remain nearly identical despite directional variations in the grating architecture, underscoring the dominant role of film conductivity anisotropy over structural orientation in determining macroscopic electrochemical behavior (**Figure S7**).

SI S7. Anisotropic electrostatic and conductive behaviors of LIPSS


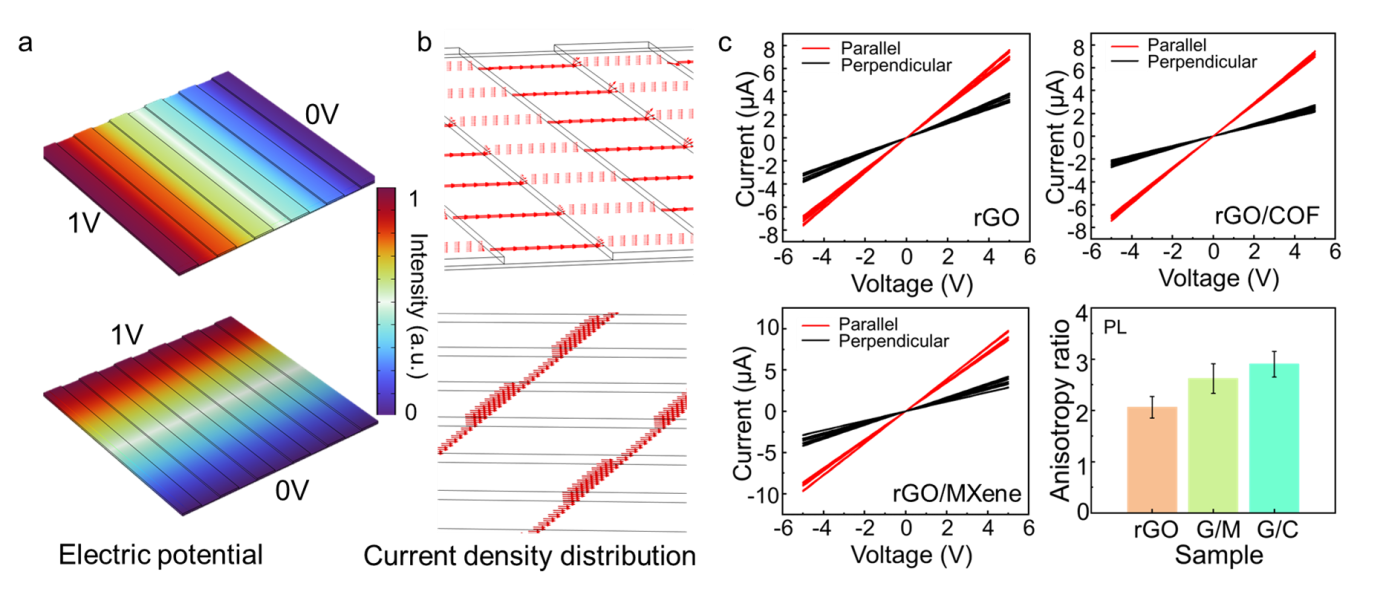


**Figure S7. Anisotropic electrostatic and conductive behaviors of LIPSS.** a-b) Simulated potential distributions and corresponding current density in the sample when the applied electric field direction is perpendicular or parallel to the grating direction. Experimental and theoretical simulation verification of anisotropic conductivity of SEP-MSC devices. c) Experimental test results of anisotropic conductivity of the graphene hybrid samples.

Finite element analysis was employed to investigate charge transport mechanisms in anisotropic micro/nanostructured rGO materials under electric fields oriented perpendicular and parallel to the gratings. Computational results revealed that the fields perpendicular to the gratings induced disordered and fragmented current pathways due to structural confinement effects, whereas parallel fields generated channelized conduction pathways with low resistance. This underscores the structural anisotropy’s role in directing charge migration, demonstrating significantly enhanced transport efficiency along the preferred orientation.

To verify this theoretical expectation, we performed multiple independent measurements at various positions on our graphene composite films featuring a surface micro/nanograting structures, as shown in **Figure S7c**. For rGO films, the results revealed a conductivity anisotropy ratio of ~2.06, with a standard deviation maintained within ±0.21, underscoring the high repeatability and stability of our measurements. Meanwhile, the rGO/COF hybrid exhibited a conductivity anisotropy ratio of 2.90±0.25, and the rGO/MXene hybrid showed 2.62±0.29. The observed anisotropy in these experiments is consistent well with the theoretical expectations, while the discrepancy might be due to the variations, such as variations in the average free path of electrons across different conductive materials, as well as deviations between experiments and theoretical predictions during processing (e.g., reduction degree of the film and surface morphology irregularities). Furthermore, in order to maintain the consistency of the text description, only the theoretical simulation results are retained in the main text.

SI S8. Electrochemical characterizations of the SEP-MSC_U_


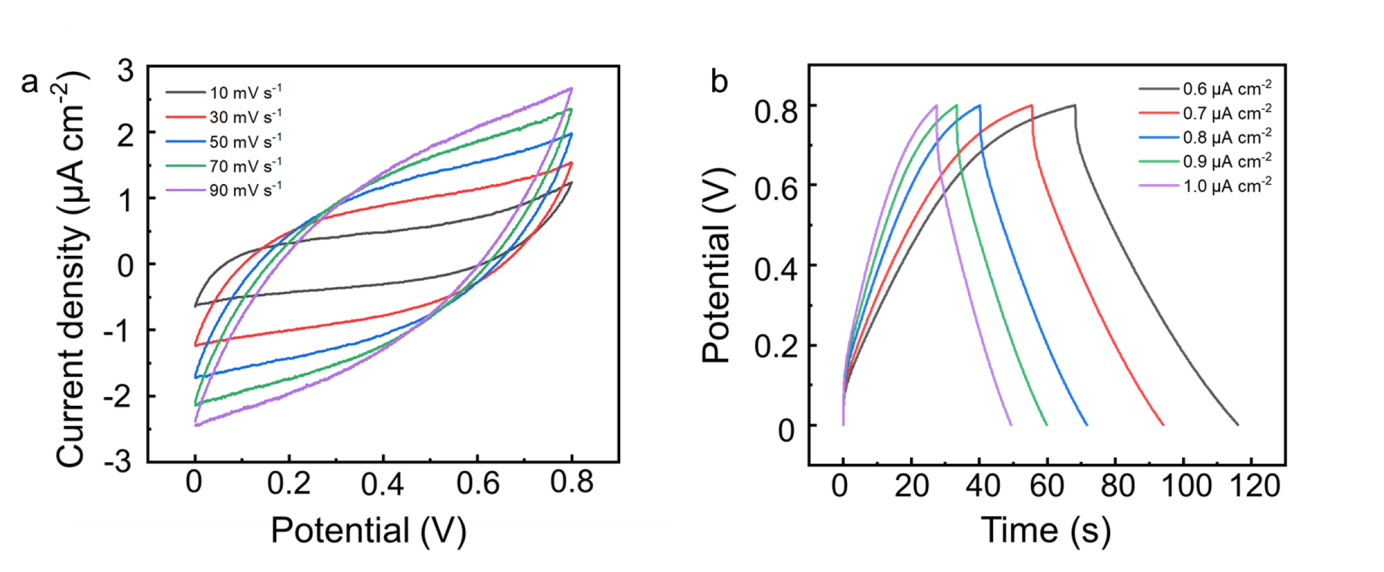


**Figure S8. Electrochemical characterizations of the SEP-MSC_U._** a) CV curves of the SEP-MSC_U_ at various scan rates. b) GCD curves at different current densities.

SI S9. LIPSS processing of different samples with SLM-FPL technology


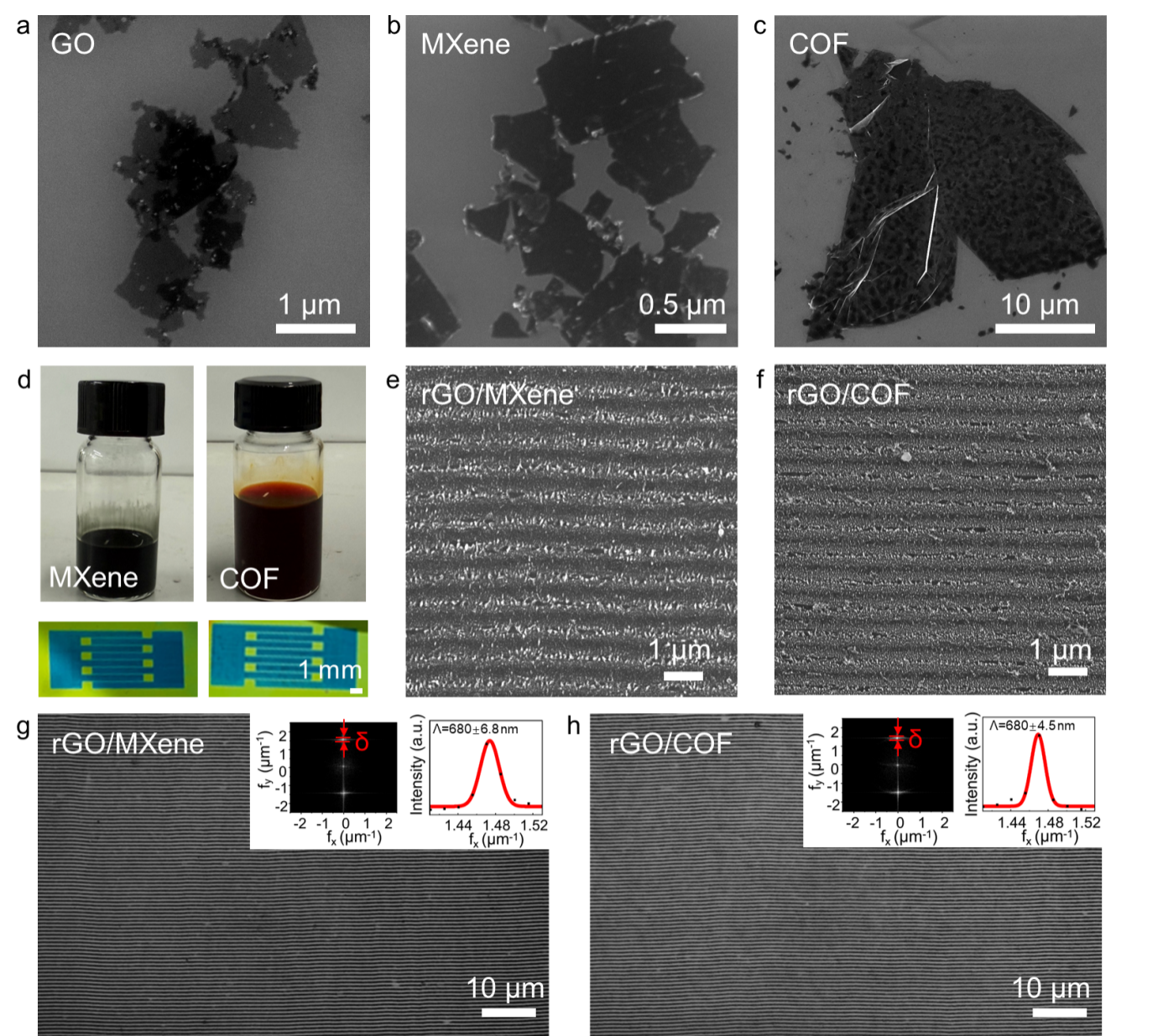


**Figure S9. LIPSS processing of different samples with SLM-FPL technology**. a-c) SEM images of dispersed GO, MXene, and COF nanosheets, respectively. d) Photographs of MXene and COF solutions, with corresponding device images enabled with SLM-FPL technology. e-f) Surface morphology characterizations of LIPSS on rGO/MXene and rGO/COF films. g-h) Large-area surface morphology characterization of rGO/MXene and rGO/COF devices. The 2D fast Fourier transform (2D-FFT) spectra show the structure periods are respectively 680±6.8 nm and 680±4.5 nm, indicating good uniformity of gratings over a large area.

SI S10. XPS compassions of composite films before and after laser processing


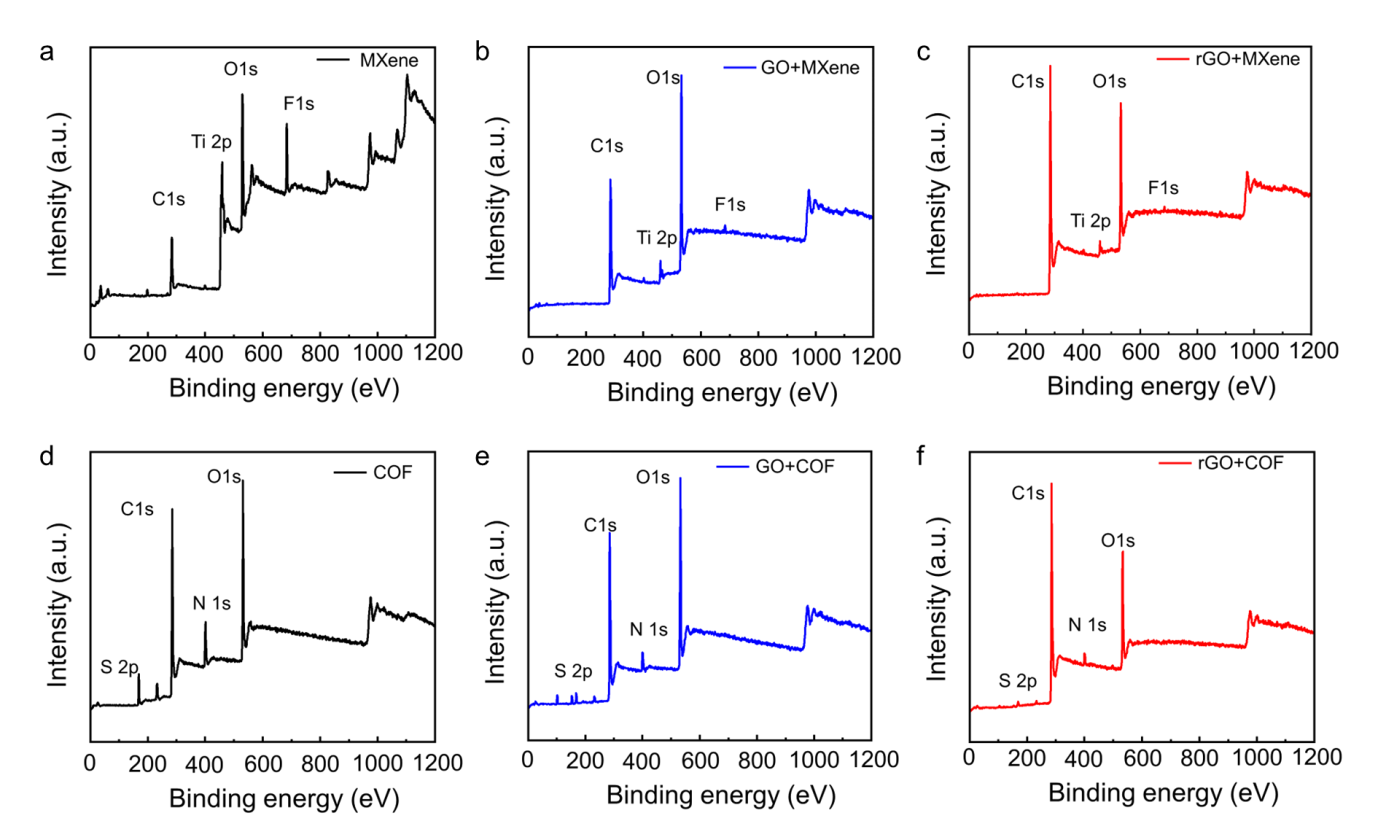


**Figure S10. XPS spectra of composite films before and after laser processing.** a–c) XPS spectra of pristine MXene (a), GO/MXene mixture (b), and laser-processed rGO/MXene (c). d–f) XPS spectra of pristine COF (d), COF/GO mixture (e), and laser-processed rGO/COF/GO (f).

SI S11. Electrochemical performance comparison of the MSC_PL_ Devices


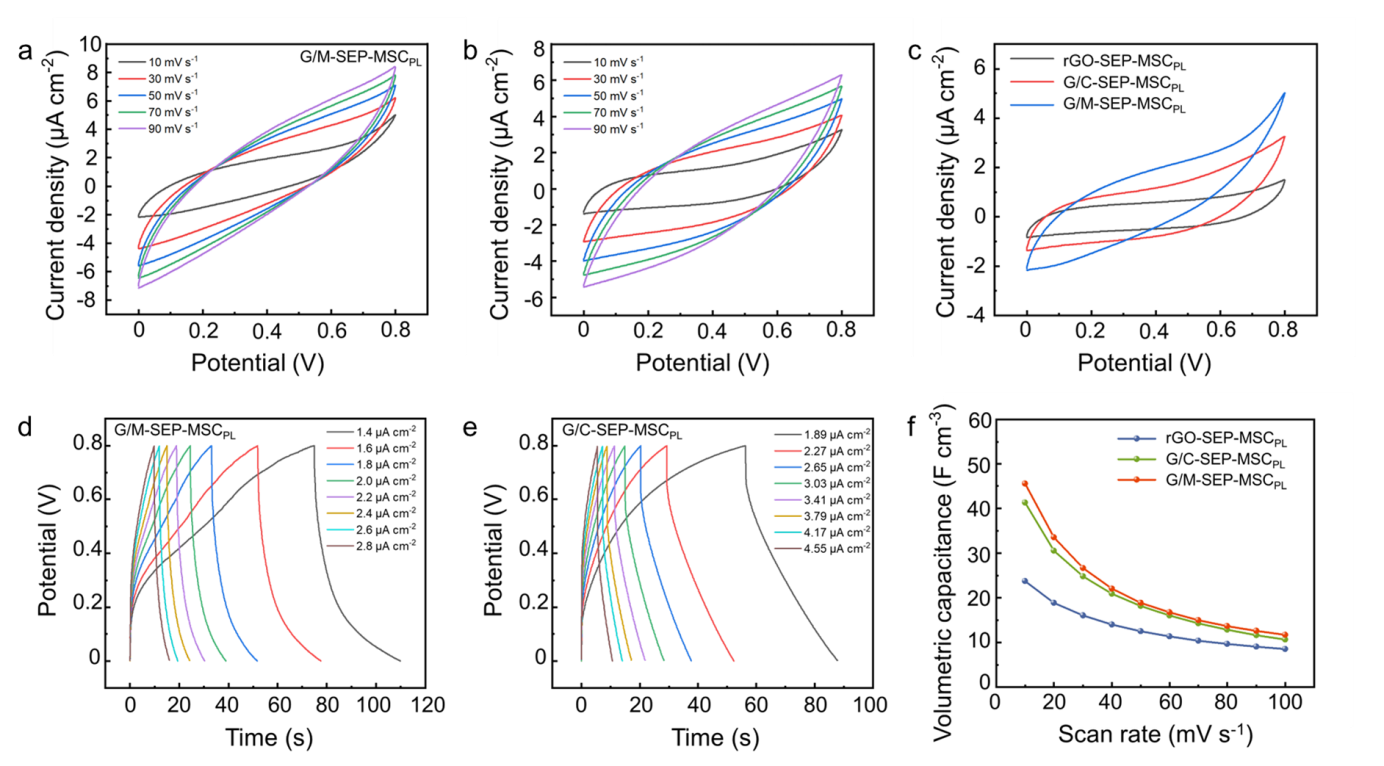


**Figure S11. Electrochemical performance comparison of the MSC_PL_ Devices.** a-b) GCD curves of G/M-SEP-MSC_PL_ and G/C-SEP-MSC_PL_ devices at different current densities. c) GCD curves of rGO-SEP-MSC_PL_, G/M-SEP-MSC_PL_, and G/C-SEP-MSC_PL_ devices at a scan rate of 10 mV s^-1^. d-e) CV curves of G/M-SEP-MSC_PL_ and G/C-SEP-MSC_PL_ devices under different scan rates. f) Comparison of volumetric capacitance for the three devices under different scan rates. Notably, the G/M-SEP-MSC_PL_, G/C-SEP-MSC_PL_ and rGO-SEP-MSC_PL_ represent the SEP-MSC_PL_ devices made of rGO/MXene, rGO/COF and rGO, respectively.

**References**

1. T. Zou, B. Zhao, W. Xin, et al., High-speed femtosecond laser plasmonic lithography and reduction of graphene oxide for anisotropic photoresponse. Light Sci. Appl., 2020, 9(1), 69.
2. B. Öktem, I. Pavlov, S. Ilday, et al., Nonlinear laser lithography for indefinitely large-area nanostructuring with femtosecond pulses. Nat. Photonics., 2013, 7(11), 897-901.
3. X. Zheng, B. Jia, H. Lin, et al., Highly efficient and ultra-broadband graphene oxide ultrathin lenses with three-dimensional subwavelength focusing. Nat. commun., 2015, 6(1), 8433.
4. L. Pei, P. Zhuang, Y. Sun, et al., Nanosupercapacitors with fractal structures: searching designs to push the limit. J. Mater. Chem. A, 2021, 9(32), 17400-17414.
